# Diversity of acoustic tracheal system and its role for directional hearing in crickets

**DOI:** 10.1186/1742-9994-10-61

**Published:** 2013-10-17

**Authors:** Arne KD Schmidt, Heiner Römer

**Affiliations:** 1 Department of Zoology, Karl-Franzens-University, Universiteatsplatz 2, Graz 8010, Austria

**Keywords:** Directional hearing, Cricket, Acoustic tracheal system, Sound localization, Interaural intensity difference (IID), Pressure difference receiver

## Abstract

**Background:**

Sound localization in small insects can be a challenging task due to physical constraints in deriving sufficiently large interaural intensity differences (IIDs) between both ears. In crickets, sound source localization is achieved by a complex type of pressure difference receiver consisting of four potential sound inputs. Sound acts on the external side of two tympana but additionally reaches the internal tympanal surface via two external sound entrances. Conduction of internal sound is realized by the anatomical arrangement of connecting trachea. A key structure is a trachea coupling both ears which is characterized by an enlarged part in its midline (i.e., the acoustic vesicle) accompanied with a thin membrane (septum). This facilitates directional sensitivity despite an unfavorable relationship between wavelength of sound and body size. Here we studied the morphological differences of the acoustic tracheal system in 40 cricket species (Gryllidae, Mogoplistidae) and species of outgroup taxa (Gryllotalpidae, Rhaphidophoridae, Gryllacrididae) of the suborder Ensifera comprising hearing and non hearing species.

**Results:**

We found a surprisingly high variation of acoustic tracheal systems and almost all investigated species using intraspecific acoustic communication were characterized by an acoustic vesicle associated with a medial septum. The relative size of the acoustic vesicle - a structure most crucial for deriving high IIDs - implies an important role for sound localization. Most remarkable in this respect was the size difference of the acoustic vesicle between species; those with a more unfavorable ratio of body size to sound wavelength tend to exhibit a larger acoustic vesicle. On the other hand, secondary loss of acoustic signaling was nearly exclusively associated with the absence of both acoustic vesicle and septum.

**Conclusion:**

The high diversity of acoustic tracheal morphology observed between species might reflect different steps in the evolution of the pressure difference receiver; with a precursor structure already present in ancestral non-hearing species. In addition, morphological transitions of the acoustic vesicle suggest a possible adaptive role for the generation of binaural directional cues.

## Introduction

When insects communicate by sound to attract mates over some distance or to compete with rivals the two main tasks for receivers are to identify and to localize the signals. Crickets are well known for their pure tone advertisement songs with carrier frequencies (CF; i.e., the signals frequency with the greatest amount of acoustic energy) between about 2 to 10 kHz to attract receptive females [1-3]. With respect to sound localization, however, they have to solve a rather complicated biophysical problem: to exploit binaural differences in sound pressure between the two ears (interaural intensity differences; IIDs). Acoustic theory predicts significant diffraction occurring only when the ratio of body size to the wavelength of sound (l:λ) exceeds a value of 0.1 [4,5]. The small body size of crickets in relation to the relatively large wavelength of the calling song prevents the establishment of reasonable IIDs through diffraction.

Furthermore, the small interaural distance between the ears in the forelegs results in only minute interaural time differences (ITDs) in the range of only 5–23 μs (calculated from distances between ears in smallest and largest cricket species at an angle of sound incidence of 45°), so that neither of these cues appears to be available for sound localization. The apparent solution to this problem is the evolution of a sophisticated pressure difference receiver, with a rather complex anatomical arrangement (for reviews see [5-9]). The inherent directionality of cricket ears results from the fact that sound can reach the external surface of the tympanum, and in addition the internal surface via a spiracular opening at the lateral surface of the prothorax. There is also a connection to the opposite ear via a transverse trachea that appears to be most crucial for establishing high IIDs. The tracheal connection displays a thin septum in the midline, a double-membrane that is responsible for the time delay in the internal sound transmission line and thus the phase relationships of the ipsi- and contralateral sound components [10-16]. Destruction of the septum changes essential characteristics in the phonotactic behaviour [17], and reduces the amount of IIDs available for localization from 10 dB to about 2 dB [16].

Because proper phase relationships between the sound components are strongly frequency dependent, directionality of the cricket ear is also strongly tuned to a narrow frequency range. This has been shown using biophysical [16] as well as neurophysiological methods [10,18,19]. Both methods applied to the same cricket species (*Gryllus bimaculatus* De Geer) yielded similar maximal values of 8 – 10 dB IID at the best frequency of directional hearing (4.5 kHz; [16,18,20]).

The fact that the pressure difference receiver of crickets is inherently and strongly frequency tuned poses another problem to the evolution of hearing: to match the best frequency of directionality with the frequency sensitivity of the ear (which should ideally be tuned to the CF of the male calling song). In a comparison of four species of field crickets, the frequency optima of the two filters involved were not matched to each other in three of the species, with a mismatch as large as 1.2 kHz [18]. These results show that a mismatch between the sensitivity and directionality tuning is not uncommon in crickets, and an observed match (such as in *T. commodus*) appears to be the exception rather than the rule. The data suggests that independent variation of both filters is possible. During evolution each sensory task may have been driven by independent constraints, and may have evolved towards its own respective optimum.

Kostarakos et al. [18] also proposed a hypothetical evolutionary scenario, where acoustic communication in crickets may have evolved originally from a close range interaction of sender and receiver under circumstances without the necessity for sound localization. With the advent of an increased active range of the signal, females at greater distances were faced with the task of localization that was as yet not – or only poorly – implemented. Therefore specific improvements to employ a pressure-difference receiver for localization became necessary (i.e., the concept of task-punctuated evolution proposed in eye evolution [21]). But due to biophysical constraints a system for localization evolved that was tuned to a specific frequency, which was difficult to match with the sensitivity tuning. However, under a high selection pressure of species competing for the acoustic communication channel both the sharpness of tuning and directional tuning can be enhanced, its mismatch can be reduced, and maximum values of 26 dB IID can be achieved [19,22]. Thus, how does the anatomical arrangement look like in cricket species where directionality is enhanced compared to the “standard” and well studied field cricket *G. bimaculatus*?

To date, the mechanical and acoustical properties required for the auditory tracheal system to account for the observed differences of IIDs remain unknown but several structural modifications of the acoustic trachea are expected to be involved. Indeed, within the Gryllidae such anatomical variation of the tracheal apparatus has been already recognized in the early work of Ander [23]. Remarkably, Ander was aware of the fact that the acoustic vesicle with the septum should have some relevance for acoustic communication and concluded that a reduction of tegminal stridulation should be coupled with the reduction of the tracheal apparatus.

In the present study we used a comparative approach on a large number of cricket species and their allies of the suborder Ensifera to correlate differences of acoustic tracheal morphology with properties of traits related to acoustic communication in order to gain a better understanding towards the evolution of the pressure difference receiver and directional hearing.

## Results

40 ensiferan species from three different superfamilies were analysed, with the majority belonging to the Grylloidea (36 species; families: Gryllidae, Gryllotalpidae and Mogoplistidae), three species belonging to Stenopelmatoidea (family: Gryllacrididae) and one species belonging to the Rhaphidophoridea (family: Rhaphidophoridae).

**Figure 1:**
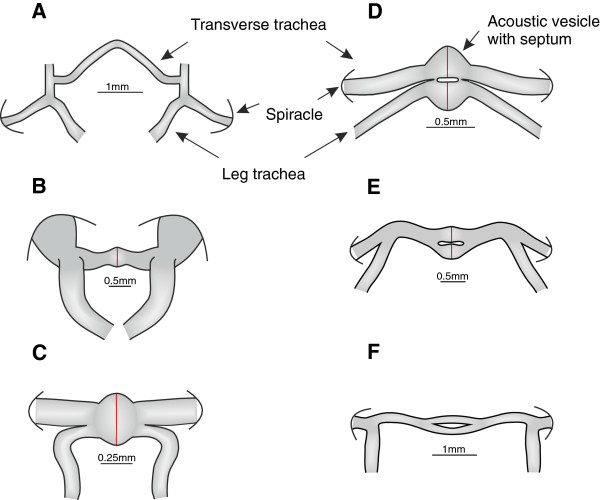
**Illustration of acoustic tracheal diversity.** Six general types of acoustic tracheal systems to illustrate their main morphological differences. **(A)** A member of the subfamily Gryllacridinae (Gryllacrididae) considered as primarily non-hearing, **(B)***Gryllus bimaculatus* (Gryllidae: Gryllinae), **(C)***Oecanthus* sp. (Gryllidae: Oecanthinae), **(D)***Paroecanthus podagrosus* (Gryllidae: Eneopterinae), **(E)***Luzara* sp. (Gryllidae: Phalangopsinae), **(F)** Phalangopsinae 1 (Gryllidae: Phalangopsinae). One of the most conspicuous features concerns modifications of the transverse acoustic trachea providing the anatomical basis for the contralateral input to the ear. The most basic form appears to be an unspecialized connecting trachea as present in the Gryllacrididae species **(A)** without a septum. Within the Gryllidae the simplest structural modification in the midline of the transverse trachea is a single, small sized vesicle as in *G. bimaculatus***(B)**. For the majority of rainforest species the acoustic vesicle is enlarged both in absolute and relative size compared with *G. bimaculatus*, or structurally modified into a double acoustic vesicle (*P. podagrosus***(D)** and *Luzara* sp. **(E)**). Tracheal system of a member of the Gryllidae subfamily Phalangopsinae characterised with secondarily loss of tibial tympana **(F)**.

Figure 1 illustrates six general types of acoustic tracheal systems emphasizing the main morphological differences encountered between species (see Additional file 1 for respective digital images). One of the most conspicuous features is related to modifications of the transverse trachea providing the anatomical basis for the respective contralateral input to the internal surface of the tympanum in the ear on both sides. The most basic form appears to be an unspecialized connecting trachea as present in the Gryllacrididae species (Figure 1A, Additional file 2) which lacks a septum. Within the Gryllidae an impressive anatomical transformation has taken place with the appearance of an acoustic vesicle. The simplest structural modification in the midline of the transverse trachea is a single, small sized vesicle as evident in the field crickets *G. bimaculatus*, *G. campestris* and *T. leo* (Figure 1B, *G. bimaculatus*). However, in many of the rainforest species the acoustic vesicle is vastly enlarged both in absolute and relative size compared with field crickets (Additional file 3). An even more striking structural modification is the appearance of a double acoustic vesicle (e.g., *P. podagrosus* and *Luzara* sp., Figure 1D and 1E, respectively). So far, in all investigated species the presence of an acoustic vesicle was always associated with a septum located along the midline of this structure. *Troglophilus neglectus*, the only member of the superfamily Rhaphidophoridea considered in this study was an exceptional case where no transverse trachea was found (Additional file 2).

In addition to the acoustic vesicle, another conspicuous morphological difference between species is the branching position of the leg trachea relative to the transverse trachea. Such a character appears to be potentially relevant in affecting sound wave interference in the tracheal system. Branching points of both trachea are highly variable; they can merge distally, closely beyond the spiracles but can also run separately and meet in the acoustic vesicle (Figure 1B and D, respectively) with various intermediate forms.

**Figure 2:**
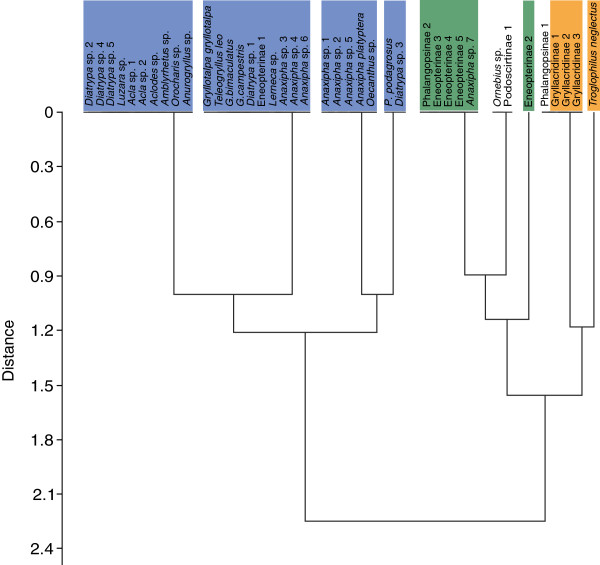
**Graphical representation of morphological/behavioural differences between species.** Dendrogram resulting from cluster analysis showing the Euclidean distance based on seven morphological/behavioural characters related to the acoustic tracheal system and directional hearing (data matrix, Additional file 2). One main cluster (in blue) comprises species that use intraspecific acoustic communication in conjunction with the possession of an acoustic vesicle. The second major cluster consists of species of the family Gryllidae that secondarily lost acoustic signalling and presumably reduced the acoustic vesicle but still have tibial ears (green cluster) and species that are primarily non-hearing (orange cluster).

A data matrix summarizes seven morphological/behavioural properties to account for differences related to the acoustic tracheal system in conjunction with the status of hearing and the use of intraspecific acoustic communication (Additional file 2). The dendrogram resulting from a cluster analysis revealed two main clusters that can be generalized in the following way: species using intraspecific acoustic communication and possess tibial tympana exhibit an acoustic vesicle and a septum (Figure 2, marked in blue). On the other hand species that either lost acoustic communication (cluster marked in green) or primarily never used acoustic advertisement calls (cluster marked in orange) lack an acoustic vesicle. Interestingly, in *Ornebius* sp. (family Mogoplistidae) and a species of the subfamily Podoscirtinae (family Gryllidae) constituting the same cluster (Figure 2) no acoustic vesicle and no septum was existent despite their ability to sing. In this respect, within the Gryllinae the strongest reduction of functional characters with complete omission of tympanal ears and a stridulatory apparatus was observed in the Phalangopsinae 1 (Figure 1F, Additional file 2).

When compared to rainforest crickets, field crickets exhibited the smallest acoustic vesicle relative to their body size (measured as pronotum width) with values ranging from 0.05 to 0.07. This ratio is thus on average 2–3 times less compared with rainforest species, for which the average ratio is 1.5 ± 0.04 (Additional file 3). On the other hand field cricket species had a more advantageous l:λ ratio with values between 0.06 and 0.11. Based on non-phylogenetic ordinary least squares (OLS) regression model we found a significant linear correlation (R^2^ = 0.37, F-test, F = 13.3, P = 0.001, N = 25) for the relationship of relative acoustic vesicle size and wavelength of calling song frequency where acoustic vesicle size tended to be larger with decreasing l:λ ratio. The significant result was corroborated when comparing with the PGLS analysis in order to account for phylogenetic history of species in this data set (Table 1).

**Table 1:** Summary of OLS and PGLS analysis

**Model**	**Coefficient**	**SE**	**t for H0**	**F for H0**	**p value**	**r**^**2**^
*OLS*						0.37
Intercept	0.196	0.018	11.047	122.034	1.131e-10	
Slope	-1.226	0.336	-3.653	13.341	0.0013	
*PGLS*						0.26
Intercept	0.171	0.032	5.278	27.859	2.346e-05	
Slope	-1.214	0.427	-2.844	8.090	0.009	

Results of the regression analysis for predicting the species acoustic vesicle size on wavelength of calling song frequency (both relative to body size) using OLS (Ordinary Least Squares; indicates conventional non-phylogenetic analysis) and PGLS (Phylogenetic Generalized Least Squares).

## Discussion

Directional hearing in crickets is achieved by an acoustic tracheal system which functions as a pressure difference receiver. The basic biophysical principles providing directionality between both ears have been described in the field cricket *Gryllus bimaculatus*[7,10,12]. The evolution of such a complicated structure has, however, received only little attention. Here we analysed acoustic tracheal morphology in various cricket species and species of other ensiferan families, and related these to the different trait expressions in acoustic signalling and hearing.

### Diversity of acoustic tracheal systems

**Figure 3:**
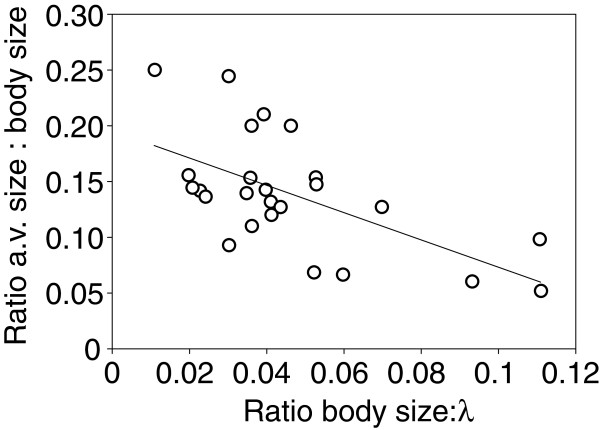
**Size correlation of the acoustic vesicle.** Relationship of the size of the acoustic vesicle (a.v.) and the wavelength (λ) of the species-specific calling song frequency, both relative to the respective body size (N = 25). Note that the analysis contained only those 25 species that exhibited an acoustic vesicle (see Additional file 2) and for which the carrier frequency of calling songs could be reliable determined based on sound recordings.

With only two exceptions (see below) the cricket species investigated in this study have been confirmed to use acoustic signals, either from evidence of direct recording of their signals or the presence of stridulatory apparatus in males. For those species, two key anatomical adaptations are documented, the acoustic vesicle and a medial septum, emphasizing their relevance for sound source localization. Most remarkable, however, was the size difference of the acoustic vesicle across the 25 species examined. In *P. podagrosus* (Figure 1D) and several other species, we found a highly enlarged acoustic vesicle consisting of a double chamber with two single central membranes, a structural differentiation markedly distinct when compared to field crickets (Additional file 2). Size variation of the acoustic vesicle alters the area of the medial septum and potentially changes the characteristics of sound transmission (e.g., phase shifting). Moreover, we observed the general trend whereby a greater deviation of the critical l:λ ratio of 0.1 towards smaller values is related to a larger relative size of the vesicle (Figure 3).

The functional role of the medial septum as phase shifter in order to derive reliable IIDs was already demonstrated by Michelsen and Löhe [16]. However, an open question remains whether and how different vesicle and septum morphology affect the generation of IIDs, and how the different magnitudes of IIDs measured in various species [16,18] are achieved. Relative septum size seems not the only attribute determining the magnitude of IIDs. In a study comparing peripheral directional properties of the field crickets *G. bimaculatus* and *G. campestris*, both exhibiting negligible differences in relative acoustic vesicle size (0.05 and 0.06 respectively; Additional file 3) a considerable difference in the amount of IIDs was demonstrated (on average 7.7 and 17.4 dB, respectively) [18]. The rainforest cricket *P. podagrosus* exhibited similar high IIDs compared with *G. campestris* (mean of 17.1 dB; [19]), yet exhibiting the second largest acoustic vesicle observed in the present study with acoustic vesicle to body size ratio of 0.24 (Additional file 3).

In terms of acoustic biophysics relevant time and phase shifts leading to constructive and destructive interference at the tympanal membranes of the ear will depend on numerous parameters such as cross-section and length of tracheal branches (i.e., transverse and leg trachea), their relative position to each other and the size of the acoustic vesicle with the medial septum [24]. Most interestingly but highly speculative in this respect is the question how sound waves will behave in a complex arrangement consisting of a double acoustic vesicle such as seen in *P. podagrosus.* Such a system will be characterized by two independent phase shifts occurring when sound is transmitted via two distinct paths to travel from the contralateral to the ipsilateral tympanum (Additional file 4). However, preliminary neurophysiological results revealed that when removing either of both pathways IIDs were reduced by 50% suggesting that the two pathways work together to generate enhanced sound transmission.

How all these different configurations observed in various species alter the sound transmission and eventually affect the binaural directional properties is not yet known and needs further investigation ideally combining physiological, biomechanical and computational methods, in order to model sound flow characteristics in these tracheal tubes.

### Evolution of acoustic communication and the pressure difference receiver

Insect ears evolved independently at least 17 times and presumably derived from mechanoreceptive chordotonal organs primarily involved in the context of proprioception and/or vibrational sensitivity [25,26]. The phylogeny of ensiferan families and the origin of acoustic communication is not solved and highly controversial with two contradictive scenarios proposed [27]. The most parsimonious scenario based on morphological and behavioural characters concludes a convergence hypothesis in the appearance of tegminal stridulation and tibial ears of at least two times [28,29]. However, according to molecular data of three ribosomal loci a monophyletic ancestral origin of acoustic communication in Ensifera is favoured [30]. Comparative neuroanatomy and analysis of neuronal elements of the vibration-sensitive tibial organ of the atympanate Rhaphidophoridae strongly supports the view that non-hearing in this group is the ancestral condition and the sensory elements regarded as precursors for audition ([26,31]; convergence hypothesis: [32]).

The atympanate Gryllacrididae are also considered as primarily non-hearing; however, in contrast to the Rhaphidophoridae they already possess a sensory organ homologue to the *crista acoustica* (a sound sensitive hearing organ found in Tettigoniidae, Haglidae, and Anostostomatidae) which is regarded as a precursor organ in the evolution of acoustic communication [26]. Based on the main differences between these two families it was concluded that the Rhaphidophoridae represent an even more primitive group with respect to the evolution of hearing compared to Gryllacrididae [32].

Based on this evidence the convergence hypothesis of the origin of acoustic communication in Ensifera receives stronger support and implies that the morphological characteristics of the acoustic tracheal system we described here for the Gryllacrididae and Rhaphidophoridae constitutes the basal situation in the evolution of pressure difference receiver (Figure1A; Additional file 2). Interestingly, the major difference between both families is the complete lack of a transverse trachea in the Rhaphidophoridae, whereas an unmodified simple connection can be already found in the Gryllacrididae. This result would be fully consistent with the evolutionary stage of hearing proposed by Strauss and Lakes-Harlan [26], considering the Rhaphidophoridae as the more primary group.

In crickets acoustic communication most likely started at close range within close proximity of male and female [33]. As a result, a long distance communication system required the evolution of directional sensitivity. Thus, a hypothetical evolutionary scenario for the appearance of the pressure difference receiver can be drawn involving at least two steps. First, the appearance of a transverse trachea interconnected with both spiracles and the leg trachea forming a tubal system imperfectly working as a simple pressure difference receiver by producing only limited directional cues. In a second step, the modification and transition of the transverse trachea towards an acoustic vesicle with a medial septum might have been the necessary step to fine tune the preexisting system in order to generate sufficient high IIDs.

Alternatively, considering a single origin of sound signaling and hearing as proposed by Jost and Shaw [30] several subsequent reductions of functional characters (hearing organs, stridulating apparatus) across different taxa would have succeeded including the acoustic tracheal structures observed in *T. neglectus* (Rhaphidophoridae) and three species of Gryllacrididae (see Additional file 2). Such character loss has also frequently occurred within Gryllidae subfamilies, as discussed below.

### Reduction of non-functional characters

Within the family Gryllidae the reduction of tegminal and hearing organs is quite common and took place several times [34,35]. Our comparative approach clearly showed that a secondary loss of acoustic signalling within the Gryllidae was always associated with the absence of an acoustic vesicle and a septum (with one exception, Eneopterinae 3; Additional file 2). Character loss and vestigialization of non-functional structures were observed across many different taxa and are explained in the light of relaxed stabilising selection for a certain trait with a strong argument of energy and material conservation associated with its reduction [36-38]. Indeed, at least for the majority of rainforest species of the family Gryllidae the acoustic vesicle takes up a considerable amount of space within the thorax. The transverse trachea with the acoustic vesicle was accompanied with a strong reduction of its primarily respiratory function [23,39]. A similar argument of a trade-off between space for the acoustic trachea in Tettigoniids (which provides higher sensitivity for hearing) and the requirement for flight muscles in the thorax has been discussed by Bailey and Kamien [40].

Localisation of mates in a complex 3-dimensional cluttered environment like the tropical rainforest might impose a relatively strong selection force on maintaining such an elaborated structure to work properly. The omission of both acoustic communication and the necessity of mate localisation could lead to regression of the vesicle (for reduction of acoustic trachea in the tettigoniid Phasmodes see [38,41]). In our study the genus *Anaxipha* provides a convincing example of the reduction hypothesis. Secondary loss of acoustic communication in *Anaxipha* sp.7 (Trigonidiinae) was accompanied with loss of the acoustic vesicle and a reduction of the overall size of the transverse trachea when compared with species of the same genus for which acoustic signalling has been demonstrated (see Additional file 2). Similarly, within the subfamily Phalangopsinae the species Phalangopsinae 1 secondarily lost tibial tympana and the stridulatory apparatus, which is accompanied with an unstructured bifurcating transverse trachea without acoustic vesicles (Figure 1F, see also Figure 2 and Additional file 2 for such examples of reduction).

However, we also found a species of rainforest cricket, *Ornebius* sp. (family Mogoplistidae), in which the connecting trachea had no acoustic vesicle and septum at all, despite the ability of males to produce calling songs. Thus, this species represents an interesting case to examine how directionality is achieved without an apparent structure which appears to be necessary for a pressure difference receiver. The l:λ ratio for *Ornebius* is only 0.05 and thus clearly disadvantageous to provide sufficient IID’s via diffraction. Even for the relatively large species *G. bimaculatus* with a l:λ ratio of 0.09, IIDs of only 1 – 2 dB have been measured when the septum was destroyed and relevant phase shifts diminished [16]. Following the currently accepted concept of tracheal biophysics in crickets it seems not very likely that *Ornebius* sp. can exploit any relevant directional cues for sound source localization. It is worthwhile to verify if the acoustic tracheal condition observed in the rainforest species *Ornebius* sp. is rather common and perhaps a distinct phylogenetic feature of this family.

Within the Gryllidae one member of the subfamily Podoscirtinae was also characterised by a lack of an acoustic vesicle and the medial septum. Acoustic signalling for the investigated species is expected since males possess typical tegminal structures such as a stridulatory file, mirror and harp (so far no calling songs of this species are available). The middle part of the transverse trachea appeared markedly narrowed but adjoined together via a small connection (appearing when the left and right tracheal branches were carefully pulled apart). Despite the lack of a medial septum the way both tracheal branches linked together could represent a quasi-coupled system and may induce some phase shift. If this situation represents an alternative way to induce phase shifts or reflects an already degenerating system of acoustic communication cannot be answered at this point and clearly needs more attention in future studies.

## Conclusions

Directional hearing in crickets requires a sophisticated arrangement of acoustic trachea. Our comparative study on acoustic tracheal morphology showed that the emergence of intraspecific acoustic communication was strongly associated with the presence of an acoustic vesicle and a medial septum, whereas these morphological features were most likely reduced in species that secondarily lost acoustic signalling. Moreover, the relative size of the acoustic vesicle (and the septum) was significantly correlated with the species body size-to-wavelength ratio, indicating its importance in the evolution of sound localization.

## Methods

### Study site and animals

The study was predominantly carried out on Barro Colorado Island (BCI; 9° 9′N, 79° 51′W, Republic of Panama) between January-March 2011 and January/February 2012. Adult crickets (Grylloidea: Gryllidae and Mogoplistidae) and leaf-rolling crickets (Stenopelmatoidea: Gryllacrididae) were caught by sweep-netting and hand collection in the forest and at lights around the research station.

Field crickets were obtained either from local breeding stock (*Gryllus bimaculatus*, University of Graz, Austria; *Teleogryllus leo*, Humboldt-University Berlin, Germany, courtesy of M. Hennig) or wild caught in Graz (*Gryllus campestris*, *Gryllotalpa gryllotalpa*). Individuals of the species *Troglophilus neglectus* (Raphidophoridae, cave crickets) were collected in caves of the karst region in Slovenia (courtesy of A. Čokl). Insects were fed *ad libitum* on a diet of lettuce, apple, oats, fish flakes and water.

### Taxonomy

Tropical rainforests and especially the Central American region including the Isthmus of Panama hold a large diversity of insect species [42], as the latest extensive studies on insect and Orthopteran diversity in Panama (IBISCA, [43]), Costa Rica [44] and the Caribbean [45] revealed a surprisingly high number (number is) of new species. Like for most other neotropical insects, the taxonomy of crickets is insufficiently known. This is also true for the cricket fauna on BCI and despite solid work by Hebard [46] in the Panama Canal Zone taxonomic affiliation of many potentially new Orthopteran rainforest species (crickets and leaf-rolling crickets) has not been achieved by now. Therefore, for subsequent taxonomic determination or re-examination and subsequent genetic barcoding analysis (*sensu*[47]) we deposited voucher specimens for every species used in this study at the ZFMK (Zoologisches Forschungsmuseum Alexander Koenig, Bonn, Germany).

### Tracheal system preparation and analysis

For tracheal preparations insects were killed by freezing at -20°C. The acoustic tracheal system was dissected ventral side up and placed on a stage micrometer (10 mm, Nikon, Tokio, Japan) under a stereo microscope (Wild M10, Leica, Wetzlar, Germany or Discovery V.12 with Plan S objective, Zeiss, Oberkochen, Germany). Digital images of preparations were taken with a microscope camera (DCM510, 5 M pixels, Oplenic Optronics CO., LTD, Hangzhou, China) and subsequently analysed using image processing software ImageJ 1.4 [48]. For illustration purposes images of the acoustic tracheal system were redrawn in CorelDraw.

In order to evaluate the relationship of the size of the acoustic vesicle and the wavelength of song carrier frequencies we determined the vesicle dimension by measuring its length along the midline (viewed from above, ventral). Sound recordings were either obtained in the laboratory from isolated singing males using electret microphones (frequency range: 50–16.000Hz, LM-09, Hama, Monheim, Germany) placed near the animal and digitized with an analogue to digital converter (sampling rate: 20 kHz, PowerLab series 4/25, ADInstruments, Sydney, Australia) or directly obtained in the habitat. Habitat recordings were made using a Telinga parabolic microphone (Pro7W, Tobo, Sweden) and digitized with a recorder (sampling rate: 44.1 kHz; Marantz PDM670, D&M Holdings Inc. Kanagawa, Japan). Song frequency determination was performed using audio software CoolEdit Pro 2.0 (Adobe Systems, California, USA). A detailed description of the sound recording method and analysis can be found in Schmidt et al. [49].

The width of the pronotum was determined as a mass-independent measure of body size based on digital photo images in order calculate the value of the quotient l:λ. A total of 40 species were investigated with respect to the difference of seven morphological/behavioural characters in the context of directional hearing. These characters comprise presence/absence (yes/no) information of: tibial tympana, intraspecific acoustic communication, acoustic vesicle, septum, transverse trachea, transverse trachea disconnected at the midpoint, leg and transverse trachea merge before the acoustic vesicle. Based on a data matrix the Euclidean distance between all species pairs was computed and a cluster analysis performed (unweighted pair-group average). Statistical analysis was carried out using software PAST [50]. In only one case, a species of the subfamiliy Podoscirtinae (Additional file 2) acoustic communication was neither directly observed nor through sound recordings confirmed; instead, specializations on male tegmina (i.e., stridulation vein, harp and mirror) were used as indictor for acoustic signalling.

### Phylogenetic control and statistical analysis

Comparative data of the acoustic vesicle size and the wavelength of the average calling song frequency relative to the species body size were tested for phylogenetic signal using phylogenetically independent contrasts. We constructed a phylogenetic tree (see Additional file 5) proposed by Gwynne [28] using the software Mesquite [51]. Taxonomic position of respective Gryllidae subfamilies were adapted and complemented with the 25 species used in the regression analyses of acoustic vesicle size (Figure 3; see also Additional file 3 for respective values). Phylogenetic distances were standardized to equal branch lengths of 1. The Mesquite tree was converted into a phylogenetic variance-covariance matrix (Mesquite package PDAP). The MATLAB program Regressionv2 [52,53] was used to examine phylogenetic effects of the data set by performing a phylogenetic generalized least-squares (PGLS) regression model and results were compared to non-phylogenetic ordinary least squares (OLS) regression.

## Competing interests

Both authors declare that they have no competing interests.

## Authors’ contributions

AKDS and HR designed the study and wrote the paper. AKDS collected and analyzed the data. Both authors read and approved the final manuscript.

## Supplementary Material

Additional file 1 **Digital images of the acoustic tracheal system.** In addition to the illustration of different acoustic tracheal systems shown in Figure 1 their respective digital images are presented. (A) A member of the subfamily Gryllacridinae (Gryllacrididae), (B) *Gryllus bimaculatus* (Gryllidae: Gryllinae), (C) *Oecanthus* sp. (Gryllidae: Oecanthinae), (D) *Paroecanthus podagrosus* (Gryllidae: Eneopterinae), (E) *Luzara* sp. (Gryllidae: Phalangopsinae), (F) Phalangopsinae 1 (Gryllidae: Phalangopsinae). Bar size = 1 mm. Click here for file

Additional file 2 **Summary of morphological/behavioural traits of 40 orthopteran species.** Data matrix of seven morphological/behavioural characters related to acoustic tracheal system in the context of directional hearing. Character description: presence (1), absence (0), not known (?). **a** tibial tympana (1/0), **b** intraspecific acoustic communication (1/0), **c** acoustic vesicle (1/2/0), **d** central membrane (septum) (1/0), **e** transverse trachea (1/0), **f** transverse trachea disconnected at the midpoint (1/0), **g** leg and transverse trachea merge before the acoustic vesicle/midline (1/0). Abbreviation of family names: GR = Gryllidae; GT = Gryllotalpidae; MP = Mogoplistidae; GT = Gryllotalpidae; GA = Gryllacrididae; RH = Rhaphidophoridae. Click here for file

Additional file 3 **Data used for the correlation of the acoustic vesicle size.** Summary of values obtained for acoustic vesicle size and the wavelength of species-specific average calling song frequency, both relative to the species body size. Comparative data were used for the analysis shown in Figure 3. Mean values of male carrier frequency (f_c_) for the following species were taken from the literature: *G. campestris* and *G. bimaculatus*[18]; *T. leo*[54]; *G. gryllotalpa*, [55]. Click here for file

Additional file 4 **Sound transmission in an acoustic tracheal system.** Schematic illustration of the ipsilateral (red arrows) and contralateral (blue arrows) sound path in an acoustic tracheal system (*P. podagrosus*) consisting of a double acoustic vesicle. Note in contrast to a single acoustic vesicle two alternative pathways from the contralateral side arise and may affect sound transmission. Click here for file

Additional file 5 **Phylogenetic tree of Gryllidae subfamilies.** Phylogenetic relationship of 25 cricket species used for comparative analysis of acoustic vesicle size (Figure 3) in order to test for phylogenetic signal. We used a phylogenetic tree of Gryllidae subfamilies proposed by Gwynne [28] and assigned our 25 species accordingly. Click here for file
