# Supplementary figures and images for: Diversity of acoustic tracheal system and its role for directional hearing in crickets
Source: Front Zool. 2013 Oct 17;10:61. doi: 10.1186/1742-9994-10-61 (PMC3852832; doi:10.1186/1742-9994-10-61)

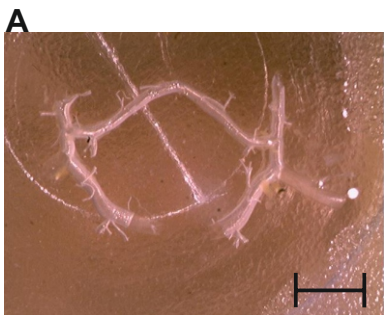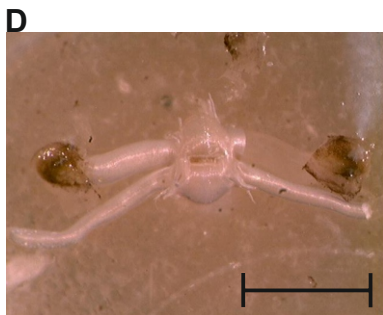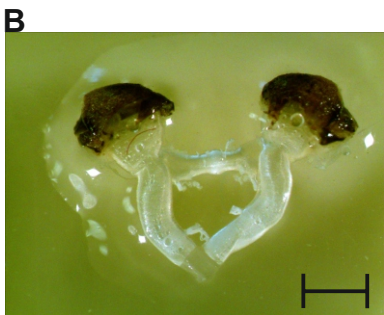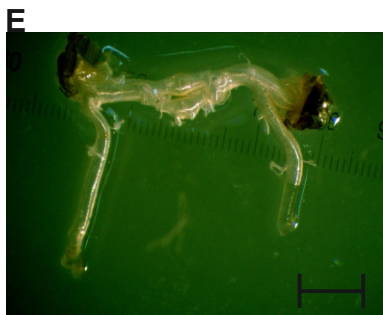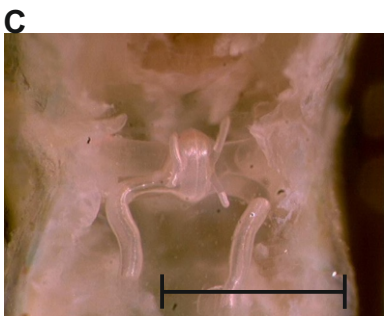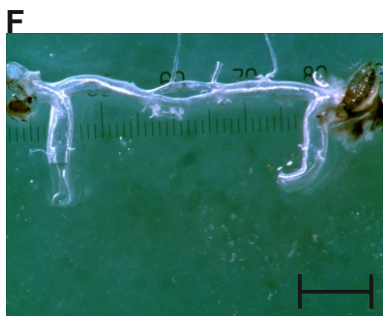

Supplement: Additional file 1 — Digital images of the acoustic tracheal system. In addition to the illustration of different acoustic tracheal systems shown in Figure 1 their respective digital images are presented. (A) A member of the subfamily Gryllacridinae (Gryllacrididae), (B) Gryllus bimaculatus (Gryllidae: Gryllinae), (C) Oecanthus sp. (Gryllidae: Oecanthinae), (D) Paroecanthus podagrosus (Gryllidae: Eneopterinae), (E) Luzara sp. (Gryllidae: Phalangopsinae), (F) Phalangopsinae 1 (Gryllidae: Phalangopsinae). Bar size = 1 mm. [file 1742-9994-10-61-S1.pdf]

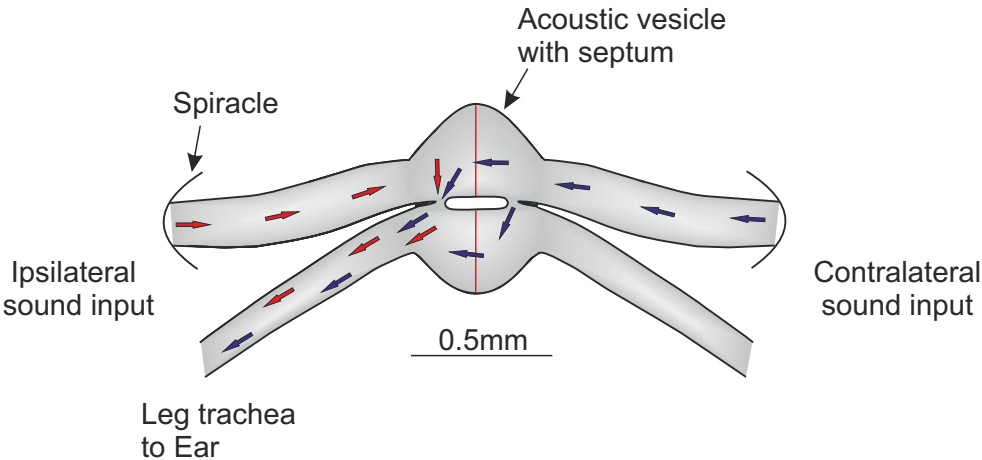

Supplement: Additional file 4 — Sound transmission in an acoustic tracheal system. Schematic illustration of the ipsilateral (red arrows) and contralateral (blue arrows) sound path in an acoustic tracheal system (P. podagrosus) consisting of a double acoustic vesicle. Note in contrast to a single acoustic vesicle two alternative pathways from the contralateral side arise and may affect sound transmission. [file 1742-9994-10-61-S4.pdf]

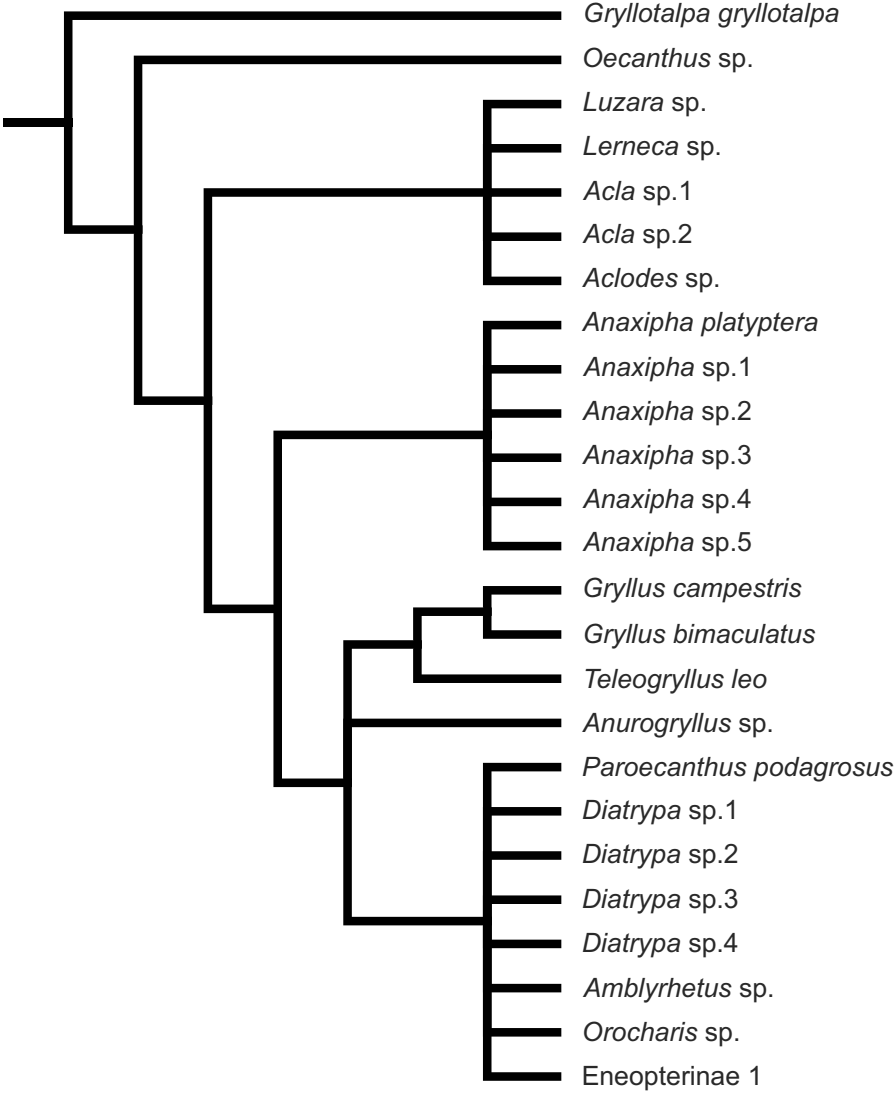

Supplement: Additional file 5 — Phylogenetic tree of Gryllidae subfamilies. Phylogenetic relationship of 25 cricket species used for comparative analysis of acoustic vesicle size (Figure 3) in order to test for phylogenetic signal. We used a phylogenetic tree of Gryllidae subfamilies proposed by Gwynne [28] and assigned our 25 species accordingly. [file 1742-9994-10-61-S5.pdf]
