# Supplementary material for: Diversity of acoustic tracheal system and its role for directional hearing in crickets
Source: Front Zool. 2013 Oct 17;10:61. doi: 10.1186/1742-9994-10-61 (PMC3852832; doi:10.1186/1742-9994-10-61)
Supplement: Additional file 2 — Summary of morphological/behavioural traits of 40 orthopteran species. Data matrix of seven morphological/behavioural characters related to acoustic tracheal system in the context of directional hearing. Character description: presence (1), absence (0), not known (?). a tibial tympana (1/0), b intraspecific acoustic communication (1/0), c acoustic vesicle (1/2/0), d central membrane (septum) (1/0), e transverse trachea (1/0), f transverse trachea disconnected at the midpoint (1/0), g leg and transverse trachea merge before the acoustic vesicle/midline (1/0). Abbreviation of family names: GR = Gryllidae; GT = Gryllotalpidae; MP = Mogoplistidae; GT = Gryllotalpidae; GA = Gryllacrididae; RH = Rhaphidophoridae. [file 1742-9994-10-61-S2.pdf]

**Family**	**Subfamily**	**Species**	**a**	**b**	**c**	**d**	**e**	**f**	**g**
GR	Gryllinae	*Gryllus bimaculatus*	1	1	1	1	1	1	1
GR	Gryllinae	Gryllus campestris	1	1	1	1	1	1	1
GR	Gryllinae	*Teleogryllus leo*	1	1	1	1	1	1	1
GR	Gryllinae	*Anurogryllus* sp.	1	1	2	1	1	1	1
GR	Phalangopsinae	*Acla* sp. 1	1	1	2	1	1	1	1
GR	Phalangopsinae	*Acla* sp. 2	1	1	2	1	1	1	1
GR	Phalangopsinae	*Aclodes* sp.	1	1	2	1	1	1	1
GR	Phalangopsinae	*Luzara* sp.	1	1	2	1	1	1	1
GR	Phalangopsinae	*Lerneca* sp.	1	1	1	1	1	1	1
GR	Phalangopsinae	Phalangopsinae 1	0	0	0	0	1	0	1
GR	Phalangopsinae	Phalangopsinae 2	1	?	0	0	1	?	1
GR	Eneopterinae	*Diatrypa* sp. 1	1	1	1	1	1	1	1
GR	Eneopterinae	*Diatrypa* sp. 2	1	1	2	1	1	1	1
GR	Eneopterinae	*Diatrypa* sp. 3	1	1	2	1	1	1	0
GR	Eneopterinae	*Diatrypa* sp. 4	1	1	2	1	1	1	1
GR	Eneopterinae	*Diatrypa* sp. 5	1	1	2	1	1	1	1
GR	Eneopterinae	*Amblyrhetus* sp.	1	1	2	1	1	1	1
GR	Eneopterinae	*Orocharis* sp.	1	1	2	1	1	1	1
GR	Eneopterinae	*Paroecanthus podagrosus*	1	1	2	1	1	1	0
GR	Eneopterinae	Eneopterinae 1	1	1	1	1	1	1	1
GR	Eneopterinae	Eneopterinae 2	1	0	0	1	1	1	1
GR	Eneopterinae	Eneopterinae 3	1	0	0	0	1	1	1
GR	Eneopterinae	Eneopterinae 4	1	0	0	0	1	1	1
GR	Eneopterinae	Eneopterinae 5	1	0	0	0	1	1	1
GR	Trigonidiinae	*Anaxipha platyptera*	1	1	1	1	1	0	0
GR	Trigonidiinae	*Anaxipha* sp. 1	1	1	1	1	1	1	0
GR	Trigonidiinae	*Anaxipha* sp. 2	1	1	1	1	1	1	1
GR	Trigonidiinae	*Anaxipha* sp. 3	1	1	1	1	1	1	1
GR	Trigonidiinae	*Anaxipha* sp. 4	1	1	1	1	1	1	1
GR	Trigonidiinae	*Anaxipha* sp. 5	1	1	1	1	1	1	0
GR	Trigonidiinae	*Anaxipha* sp. 6	1	1	1	1	1	1	1
GR	Trigonidiinae	*Anaxipha* sp. 7	1	0	0	0	1	?	1
GR	Oecanthinae	*Oecanthus* sp.	1	1	1	1	1	1	0
GR	Podoscirtinae	Podoscirtinae 1	1	0	0	1	1	1	1
MP	Mogoplistinae	*Ornebius* sp.	1	0	0	1	1	1	1
GT	Gryllotalpinae	*Gryllotalpa gryllotalpa*	1	1	1	1	1	1	1
GA	Gryllacridinae	Gryllacridinae 1	0	0	0	0	1	0	1
GA	Gryllacridinae	Gryllacridinae 2	0	0	0	0	1	0	1
GA	Gryllacridinae	Gryllacridinae 3	0	0	0	0	1	0	1
RH	Troglophilinae	*Troglophilus neglectus*	0	0	0	0	0	-	-
