# Supplementary material for: Diversity of acoustic tracheal system and its role for directional hearing in crickets
Source: Front Zool. 2013 Oct 17;10:61. doi: 10.1186/1742-9994-10-61 (PMC3852832; doi:10.1186/1742-9994-10-61)
Supplement: Additional file 3 — Data used for the correlation of the acoustic vesicle size. Summary of values obtained for acoustic vesicle size and the wavelength of species-specific average calling song frequency, both relative to the species body size. Comparative data were used for the analysis shown in Figure 3. Mean values of male carrier frequency (fc) for the following species were taken from the literature: G. campestris and G. bimaculatus[18]; T. leo[54]; G. gryllotalpa, [55]. [file 1742-9994-10-61-S3.pdf]

Species	Mean male f_c (kHz)	Ratio	
		Vesicle size:Body size	Body size:Wavelength
Gryllus campestris	4.7	0.05	0.11
Gryllus bimaculatus	4.7	0.06	0.09
Telegryllus leo	3.0	0.07	0.06
Gyllotalpa gryllotalpa	1.6	0.07	0.05
Lerneca sp.	4.2	0.09	0.03
Anurogryllus sp	7.0	0.10	0.11
Eneopterinae 1	6.2	0.11	0.04
Anaxipha sp. 2	7.1	0.12	0.04
Diatrypa sp. 1	4.0	0.13	0.04
Orocharis sp.	5.3	0.13	0.07
Acla sp. 2	3.2	0.13	0.04
Anaxipha sp. 3	7.5	0.14	0.02
Acla sp. 1	3.1	0.14	0.03
Anaxipha sp. 1	6.5	0.14	0.02
Luzara sp.	2.9	0.14	0.04
Anaxipha sp. 4	7.9	0.14	0.02
Aclodes sp.	4.8	0.15	0.05
Anaxipha sp. 5	8.2	0.15	0.04
Amblyrhetus sp.	4.4	0.15	0.05
Anaxipha platyptera	7.5	0.16	0.02
Diatrypa sp. 3	5.4	0.20	0.04
Diatrypa sp. 5	6.2	0.20	0.05
Diatrypa sp. 4	5.5	0.21	0.04
Paroecanthus podagrosus	3.8	0.24	0.03
Oecanthus sp.	2.7	0.25	0.01
